# Quantitative Elasticity Mapping of Submicron Silica Hollow Particles by PeakForce QNM AFM Mode

**DOI:** 10.3390/nano13131916

**Published:** 2023-06-23

**Authors:** Dmitry R. Streltsov, Kirill M. Borisov, Aleksandra A. Kalinina, Aziz M. Muzafarov

**Affiliations:** 1 Enikolopov Institute of Synthetic Polymeric Materials of Russian Academy of Sciences, 117393 Moscow, Russia; borisov@ispm.ru (K.M.B.); kalinina@ispm.ru (A.A.K.); aziz@ispm.ru (A.M.M.); 2 A.N. Nesmeyanov Institute of Organoelement Compounds of Russian Academy of Sciences, 119334 Moscow, Russia

**Keywords:** elasticity mapping, Young’s modulus, nanomechanics, silica hollow particles, buckling instability, atomic force microscopy, force spectroscopy

## Abstract

Silica hollow spheres with a diameter of 100–300 nm and a shell thickness of 8±2 nm were synthesized using a self-templating amphiphilic polymeric precursor, i.e., poly(ethylene glycol)-substituted hyperbranched polyethoxysiloxane. Their elastic properties were addressed with a high-frequency AFM indentation method based on the PeakForce QNM (quantitative nanomechanical mapping) mode enabling simultaneous visualization of the surface morphology and high-resolution mapping of the mechanical properties. The factors affecting the accuracy of the mechanical measurements such as a local slope of the particle surface, deformation of the silica hollow particles by a solid substrate, shell thickness variation, and applied force range were analysed. The Young’s modulus of the shell material was evaluated as E=26±7 GPa independent of the applied force in the elastic regime of deformations. Beyond the elastic regime, the buckling instability was observed revealing a non-linear force–deformation response with a hysteresis between the loading and unloading force–distance curves and irreversible deformation of the shell at high applied forces. Thus, it was demonstrated that PeakForce QNM mode can be used for quantitative measurements of the elastic properties of submicon-sized silica hollow particles with nano-size shell thickness, as well as for estimation of the buckling behaviour beyond the elastic regime of shell deformations.

## 1. Introduction

The importance of micro- and nanocapsule systems in biology, medicine, cosmetics and the food industry has continuously increased. Such systems are generally used for encapsulation and controlled release of various agents, e.g., drugs, enzymes, genes and other bioactive molecules for medical [1,2,3,4] and agricultural [5] applications, dyes for impact damage indicator coatings [6,7], healing agents for self-healing polymeric materials [8,9], flavours or fragrances for cosmetic and food applications [10,11,12]. Capsule release can be achieved whether by disrupting the solid shell membrane or controlling its permeability with external stimuli, e.g., salt concentration, temperature, pH, light, direct or adhesion-induced mechanical deformation [13,14].

Designing capsules for specific tasks requires understanding, tuning and controlling their physicochemical properties. Mechanical properties are obviously important for capsule stability. The capsules should be able to maintain integrity during their manufacturing, processing, storage and end-use applications. On the other hand, the shell membrane should not be too rigid to ensure fast and efficient release of the encapsulated substance under pre-set conditions. Mechanical properties of the capsules also affect the adhesion area between the capsules and substrate, and as a consequence their adhesive interaction [15,16]. Recently, it has been recognized that the mechanical properties of nanoparticles and nanocapsules (with size of about 100 nm) play an important role in regulating their biological functions, in particular their blood circulation time. The longer circulation time of softer nanoparticles was attributed to their superior deformability allowing them to pass through pores of smaller size than their diameter without losing structural integrity. The mechanical properties of nanoparticles also affect their biodistribution in the tissues and organs, tumour penetration, and tumour cell uptake [17,18,19], which is crucial for their applications for targeted drug delivery.

A wide range of applications results in a great variety of capsule dimensions, shell materials and consequently their mechanical properties. Several techniques have been developed recently to characterize the mechanical properties of micro- and nanocapsules [20,21]. These methods can be divided into two general categories, i.e., ensemble and single-capsule methods. In ensemble methods (e.g., the methods based on the use of shear forces in a turbine reactor [22], on a study of rheological properties of capsule suspensions [23,24,25] or on osmotic pressure experiments [14,26,27]), a batch of capsules is measured simultaneously, yielding an average value. Single-capsule methods provide more detailed information on their mechanical properties but require sequential measurements of individual capsules, which can be quite time consuming. Experiments with optical tweezers [28,29], deformation of capsules in a spinning drop apparatus [30], and micropipette aspiration [31,32] are based on the analysis of capsule shape changes under external stresses with an optical microscope. Thus, they are only suitable for relatively large microcapsules. Similar limitations are apparent for nanoindentation experiments [33]. Moreover, some of these methods are quite system-specific (e.g., they can only be used for relatively soft capsules due to low applied forces) or based on custom-made devices.

Silica is one of the promising materials for capsule shells due to its mechanical robustness, thermal and chemical stability, non-toxicity, low cost in production, optical transparency and easy functionalization [34,35,36,37]. Over the past decades, silica particles and capsules with various morphologies and dimensions have been synthesized and investigated systematically by many research groups. It should be noted that the inherent nature of the synthesis results in micro- or mesoporous structures of the silica materials fabricated by a sol–gel method. Moreover, the porosity, pore size distribution and pore structure can be tailored by varying the synthesis method or reaction conditions, e.g., pH of the reaction mixture, the characteristics of the surfactants used, as well as the concentrations and sources of silica [38,39,40]. On the other hand, the mechanical properties of porous silica materials strongly depend on their microstructure and overall degree of porosity. This effect has been thoroughly studied for bulk materials and thin films using classical indentation as well as AFM-based techniques [41,42,43,44]. However, a study of the mechanical properties of individual submicron silica particles and capsules is still challenging due to a combination of their small size and the high stiffness of silica. There are only very few studies available focused on experimental measurements of the mechanical properties of silica hollow [45,46,47] and solid [48,49,50] particles.

Recently, M. Möller et al. developed a method for one-step fabrication of meso-structured silica particles and capsules, replicating the self-assembled structures of a novel amphiphilic precursor polymer, i.e., poly(ethylene glycol) (PEG)-substituted hyperbranched polyethoxysiloxane (PEOS) (PEG-PEOS), in water [51] or at the oil–water interface in oil-in-water emulsions [52]. The advantage of this approach is that the amphiphilic silica precursor can self-assemble without the addition of surfactants into various meso-structures, that can be directly converted to meso-structured silica nanomaterials via the sol–gel process without any additives and further calcination steps. This group has also demonstrated that this technique can be employed for highly efficient encapsulation of enzymes in silica nanocapsules during their formation process [3] and preparation of adaptive hybrid capsules with microgel/silica composite walls of tunable permeability [53]. To the best of our knowledge, no studies devoted to the mechanical properties of such silica capsules have been published to date in spite of their potential applications, e.g., for targeted drug delivery.

Among the general-purpose techniques, atomic force microscopy (AFM) and uniaxial compression testing with micromanipulators are the most popular methods to study the mechanical properties of nanocapsules and nanoparticles [21,54]. Both methods utilize a fine probe, which indents the sample surface to a given deformation or rupture while recording the force–displacement curves. A typical force range for the micromanipulation technique is between a few μN and N. AFM, being initially developed as an imaging technique, can operate with much smaller indentation forces ranging from about 10 pN to a few μN [20,21], making it a more preferable tool for measuring the mechanical properties of the capsules with thin shell in elastic deformation regime, where the maximum capsule deformation is on the order of the shell thickness. An advantage of the AFM methods is the measurements of mechanical properties simultaneously with imaging, which can be used to precisely locate the probe relative to the particle, whereas integration in a scanning or transmission electron microscope is required for the measurements of submicron particles using the micromanipulation technique [48,49,55]. Moreover, the classical force–volume AFM mode [56] and recently introduced sub-resonance tapping modes (e.g., the PeakForce Tapping QNM by Bruker [57], Quantitative Imaging by JPK Instruments [58], Hybrid mode by NT-MDT [59]) allow to collect the force–displacement curves at each pixel of an AFM image, enabling high-resolution mapping of the mechanical properties.

It should be noted that the accurate quantitative AFM measurements of elasticity are still challenging [60,61,62,63,64].

1. Since the force loading and detection in AFM is based on cantilever bending, and the cantilever is inclined relative to the sample in the cantilever holder, a straight vertical indentation is impossible for the entire force range. Therefore, special care of the force range and cantilever stiffness is required for quantitative measurements of mechanical properties in AFM experiments. Moreover, because of a non-linear relationship between the detected signal and the actual deflection of the AFM cantilever, optical lever sensitivity calibration of the AFM setup should be carried out in the whole applied force range [65].
2. If the surface normal of the sample is not aligned with the vertical Z-axis of the AFM scanner, the AFM tip contacts the surface at an angle not anticipated by most contact mechanics models affecting the accuracy of the mechanical measurements [66,67]. Such samples as individual particles or capsules immobilized on a solid substrate present significant variations of the local slope (up to $90^{\circ }$) resulting in great changes in the tip–sample contact area. Moreover, non-vertical contact between the AFM tip and the sample produces lateral forces acting on the cantilever in addition to the normal force. While the normal stiffness of the tip–surface contact is usually described with the contact mechanics models, the lateral forces strongly depend on the type of contact (e.g., free sliding, pinning, friction) that occurs between the tip and the sample, which is difficult to predict. These lateral forces lead to an additional contribution to the normal deflection of the cantilever which can result in both underestimation and overestimation of the measured stiffness depending on the surface slope and AFM tip geometry [66,67]. To circumvent this issue, many studies choose regions of interest from relatively flat sections of the sample surface.
3. Another important factor is the mechanical stress related to the AFM probe apex radius. It was demonstrated that the use of sharp commercial AFM probes with a tip radius of about 20 nm led to a non-linear stress–strain response in relatively soft polymeric samples (e.g., polyurethanes, polystyrene), resulting in overestimation of the Young’s modulus [60,61]. This effect disappears when dull probes with a radius of several hundreds of nm were used. Moreover, the measured moduli were independent of the indentation depth and close to those evaluated by nanoindentation and DMA measurements when dull AFM probes were used. Similar results were reported for AFM measurements of elasticity for rigid materials (e.g., fused silica, HOPG, Si, Au) [63]. The measurements using a sharp tip with 40 nm radius resulted in a large deviation (up to 122%) of the Young’s moduli from their nominal values, whereas this deviation was only up to 13% when a blunt tip with 200 nm radius was used. The authors suggested that plastic deformations were the main reason for this difference. Regarding the mechanical property measurements of spherical shells, it was demonstrated that the use of sharp commercial AFM probes could result in puncturing of the thin silica shells at relatively low indentations, seriously limiting the applied force range [46]. Moreover, finite-element analysis of elastic shell indentation with an AFM tip of different geometry reveals an effect of the tip radius on the shape of the force–distance curves [68,69].
4. Nowadays, various contact mechanics models have been developed to evaluate the elastic modulus from force–distance measurements, e.g., the Hertz [70], Sneddon [71], Reissner [72], Johnson–Kendall–Roberts (JKR) [73], and Derjaguin–Muller–Toporov (DMT) [74] models. These models’ validity is strongly dependent on the AFM probe, sample size and geometry, material stiffness, and adhesion between the AFM probe and sample. Correct description of the contact mechanics is obviously crucial for accurate evaluation of the elastic modulus.
5. One more factor affecting the accuracy of the mechanical properties measurements of hollow and solid particles is the effect of the rigid substrate (also known as the stress-interaction effect [75]) because during indentation experiments the upper part of the particles is deformed by the AFM probe, whereas the bottom part is simultaneously deformed by the substrate [76,77]. Interestingly, if the substrate effect is not accounted for, the indentation of particles and capsules against a substrate leads to an underestimation of the material elastic modulus, in contrast to an overestimation of the mechanical properties during indentation of thin films [78].

In this work we focus on quantitative elasticity mapping of silica hollow nanoparticles prepared from the PEG-PEOS amphiphilic precursor by the PeakForce QNM AFM mode and compare these results with previously published ones for silica hollow and solid particles synthesized by the Stöber method with traditional AFM-based force spectroscopy and nanoindentation techniques. The factors affecting the accuracy of the measurements were analysed.

## 2. Materials and Methods

### 2.1. Materials

Tetraethoxysilane (99%, EKOS-1, Moscow, Russia), acetic anhydride (98%, Merck, Darmstadt, Germany), aqueous solution of ammonia (25%, SIGMATEK, Khimki, Russia), tetraethyl orthotitanate (97%, Merck, Darmstadt, Germany), poly(ethylene glycol) monomethyl ether (average molecular weight 350 g/mol, ABCR, Karlsruhe, Germany), toluene (Component-Reaktiv, Moscow, Russia), polystyrene (grade 525M, $\mathrm{MFI}=10.1\;g\;{\mathrm{dmin}}^{-1}$) (Nizhnekamskneftekhim, Nizhnekams, Russia), (3-aminopropyl) triethoxysilane (98%, ABCR, Karlsruhe, Germany).

### 2.2. Synthesis of Silica Hollow Particles

#### 2.2.1. Synthesis of PEOS

Hyperbranched polyethoxysiloxane (PEOS) was prepared via a synthetic route proposed by X. Zhu et al. [79]. Tetraethoxysilane (416 g, 2 moles) was mixed with acetic anhydride (214 g, 2.1 moles) and tetraethyl orthotitanate (1.4 g, 0.006 moles) under an inert atmosphere. The mixture was heated to $135{\;}^{\circ }C$ in an oil bath under intensive stirring. The resulting ethyl acetate was continuously distilled off. The supply of heat was continued until the distillation of ethyl acetate stopped. Afterwards, the product was dried in a vacuum to completely remove any volatile compounds. Synthesized PEOS was characterized by size-exclusion chromatography ($M_{w}=1000$ g/mol, $M_{w}/M_{n}=2.3$).

#### 2.2.2. Synthesis of PEOS-PEG

Poly(ethylene glycol) (PEG)-substituted PEOS was synthesized via a synthetic route proposed by M. Möller et al. [51]. Synthesized PEOS (58 g) was mixed with poly(ethylene glycol) monomethyl ether (26 g, $M_{w}=350$ g/mol) under intensive stirring. The mixture was heated to $135{\;}^{\circ }C$ under an argon atmosphere until the distillation of ethanol stopped. Afterwards, the resulting product was dried in a vacuum to completely remove any volatile compounds. The degree of modification was determined by ${}^{1}H$ NMR spectroscopy, namely 10.7 mol.% of ethoxy groups were replaced by PEG.

#### 2.2.3. Conversion of PEOS-PEG to Silica Hollow Particles

PEG-PEOS (4 g) was dispersed in distilled water (100 g) under stirring at 700 rpm for 1 min until formation of a uniform dispersion. Then, toluene (4 g) was added to the mixture under stirring at 700 rpm for 3 min. Afterwards, an ammonia aqueous solution (25%, 4 g) was added, and the reaction mixture was stirred for 24 h. Finally, the capsules were separated by centrifugation at 11,000 rpm for 1 h, rinsed three times with distilled water and dried in a vacuum.

### 2.3. Characterization Methods

${}^{1}$H NMR spectra were recorded on a Bruker WP250 SY spectrometer (Bremen, Germany) and a Bruker Avance AV300 spectrometer (Bremen, Germany) using tetramethylsilane as an internal standard.

Size-exclusion chromatography (SEC) analysis was carried on a chromatographic system consisting of a Staier seriya 2 high-pressure pump (Akvilon, Podolsk, Russia), a Smartline RI 2300 refractometric detector, and a Jet stream 2 Plus thermostat (Knauer, Berlin, Germany). The temperature was $+40\pm 0.1{\;}^{\circ }C$, THF or toluene + 2% THF were used as eluents, and the flow rate was $1.0\;\mathrm{mL}\;\min^{-1}$. The setup consisted of a column 300×7.8 mm packed with a Phenogel sorbent (Phenomenex, Torrance, CA, USA) with a particle size of 5 mm and pore size in the range from $10^{3}$ to $10^{5}$ Å. Calibration with linear polystyrene standards was used to estimate the molecular weight.

Transmission electron microscopy (TEM) measurements were carried out on a LEO912 AB OMEGA microscope (LEO Elektronenmikroskopie GmbH, Oberkochen, Germany) operating at accelerating voltages of 60, 80, 100, 120 kV.

### 2.4. PeakForce Tapping QNM

The AFM measurements were carried out with a Multimode 8 microscope in the sub-resonance PeakForce QNM AFM mode (Bruker Nano Inc., Santa Barbara, CA, USA) at a peak force tapping frequency of 2 kHz. A Nanoscope V controller and Nanoscope software version 8.10 were utilized. All measurements were performed under ambient conditions at room temperature. A standard cantilever holder for operation in air was used. The Peakforce Capture option was activated to collect, along with a surface topographic map (i.e., a height image), the force–displacement curves acquired at each pixel of the AFM image for offline analysis with different tip–sample interaction models. Before the experiments, the piezo-scanner of the microscope was calibrated using the standard calibration procedure.

#### 2.4.1. Sample Preparation for AFM Measurements

To prevent the particles from motion on the substrate during the AFM measurements, two methods of their immobilization were tested.

In the first method, to enhance adhesion between the clean silicon wafer and silica hollow particles, the substrate was silanized with APTES ((3-aminopropyl) triethoxysilane) (1 wt.% solution in ethanol, incubation time of 30 min followed by rising with toluene) [80]. Then, the silica particles dispersed in ethanol were deposited onto the silanized silicon wafer by spin coating (3000 rpm for 60 s) of the dispersion. Finally, the samples were dried overnight under ambient conditions.

The second method of immobilization was analogous to the one suggested by Zhang et al. [45], where silica hollow particles were partially embedded in a thin polystyrene (PS) film obtained by spin coating from toluene solution on a silicon wafer. The thickness of the PS films was measured by AFM and found to be about 40 nm. Afterwards, the silica hollow particles dispersed in ethanol were deposited onto the glassy PS film by spin coating the dispersion. Finally, the substrates were placed in an oven and kept at $150{\;}^{\circ }C$ for 1 h. At devitrification of the PS film, the capillary forces suck the silica particles into the film leading to their strong fixation onto the substrate after cooling the samples down to room temperature.

It was found that the immobilization with a thin PS film provides more reliable fixation of the silica particles compared to that with APTES, especially at high applied forces beyond the linear elastic regime of deformations. However, at low applied forces, the mechanical response of hollow particles was found to be independent of the method of their immobilization, in line with previous observations of Zhang et al. [45].

#### 2.4.2. AFM Probes

**Figure 1:**
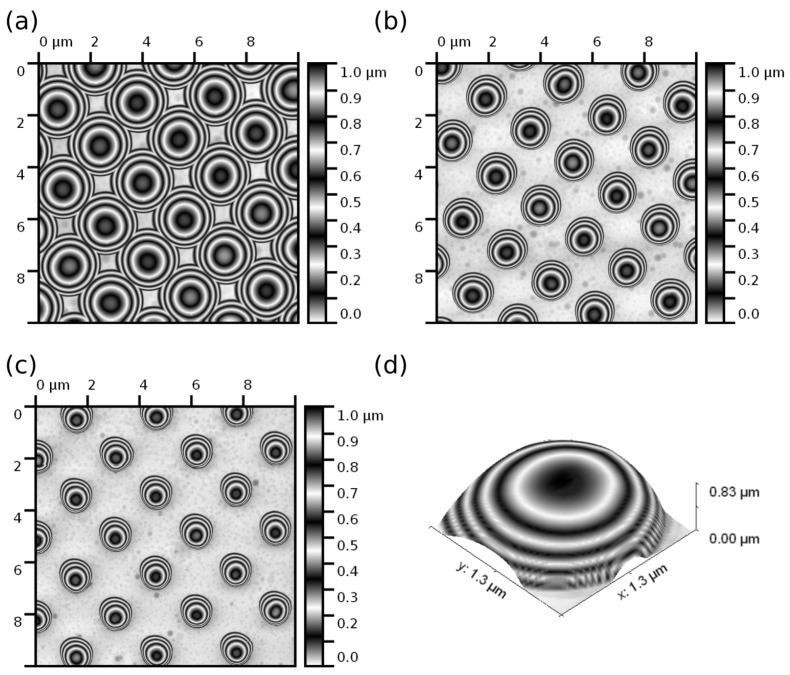
AFM height images of a reverse-imaging calibration grid sample TGT1 (NT-MDT, Moscow, Russia) obtained using the probes with a tip radius of (**a**) R=1100±50 nm (estimated tip surface RMS roughness $R_{q}=1.0\pm 0.2$ nm), (**b**) R=600±30 nm ($R_{q}=1.5\pm 0.5$ nm), (**c**) R=350±30 nm ($R_{q}=2.0\pm 0.5$ nm). Scan size is $10\times 10\;\mu m^{2}$ for all the images. (**d**) Reconstructed shape of the probe apex with a tip radius of R=600±30 nm.

All mechanical measurements were performed using specially prepared hemispherical dull probes with a tip radius of about 100 nm. The tip radius value was chosen to be smaller than the hollow particle radius to avoid the effect of the probe radius on the tip–particle contact area after buckling the shell, but high enough to minimize the contact pressure to prevent puncturing of the silica shell or breakage of the AFM probe tip. The “dull” probes were made from commercial silicon RTESP-300 probes (Bruker Nano, Inc., Santa Barbara, CA, USA) without a reflective coating with a nominal force constant of about 40 N/m by annealing in air at $1150{\;}^{\circ }C$ for 0.5–3 h [81] (variation in the annealing time allows to adjust the tip radius from several tens of nm to a few μm with a probe apex geometry close to an ideal spherical shape, cf. Figure 1a–c). The spring constant of the AFM cantilever was chosen to match the contact stiffness of the silica shell and produce sufficient indentation of the shell comparable to the cantilever deflection [82,83]. The tip radius and geometry of all the probes were evaluated by scanning a TipCheck sample (a titanium roughness sample by Bruker, Santa Barbara, CA, USA) and a reverse-imaging calibration grid sample (TGT1 by NT-MDT, Moscow, Russia). The obtained height images were processed through Nanoscope Analysis ver. 1.40 (Bruker) and Gwyddion ver. 2.60 software, both of which allow for the estimation of the tip apex radius and shape by a blind reconstruction method [84,85] (cf. Figure 1d). All probes were checked twice, i.e., before and after the AFM measurements of the silica hollow particles.

#### 2.4.3. PeakForce QNM Calibration

The spring constant of cantilevers was evaluated using the Sader method [86]. The sensitivity of the AFM photodetector (i.e., deflection sensitivity) was calibrated by ramping a cantilever against a clean sapphire calibration sample by Bruker (E=360 GPa) at a Z-ramp rate of 1 Hz at ten different points and averaging the results. The deflection sensitivity was calibrated for the whole force range used in the PeakForce QNM AFM experiments accounting for the non-linearity of the photodetector. In the PeakForce AFM mode the sample moves vertically in regard to the cantilever at a fixed frequency (e.g., 2 kHz in this study) and amplitude (the PeakForce amplitude was chosen to be low enough to collect enough data points in the tip–sample contact region, but high enough to overcome any adhesion forces, usually within the range of a few tens of nm). Both the phase (i.e., Sync. Distance) and the amplitude (i.e., Drive3 Amplitude Sensitivity) of the sample oscillations are dependent on the frequency and precise configuration of the AFM system. These parameters were calibrated at the PeakForce frequency of 2 kHz on a sapphire calibration sample (Bruker) using the high-speed data capture (HSDC) option, allowing the raw deflection signal to be captured on the AFM photodetector as a function of time.

#### 2.4.4. Automated AFM PeakForce Curves Processing

Each PeakForce Tapping AFM image (with activated the PeakForce Capture option enabling the collection of raw cantilever deflection data) contains thousands of force–displacement curves (e.g., 65,536 pairs of curves for an AFM image with a resolution of 256×256 pixels). In order to automate the mechanical data treatment for evaluation of the elastic modulus of the silica shells, a home-made Python program based on the PyUSID open-source framework for storing and analysing imaging and spectroscopy data [87] was developed. This program enables the translation from the proprietary Bruker PeakForce Capture (.PFC) file format to an open-source HDF5 format for processing of the force–distance curve data.

**Figure 2:**
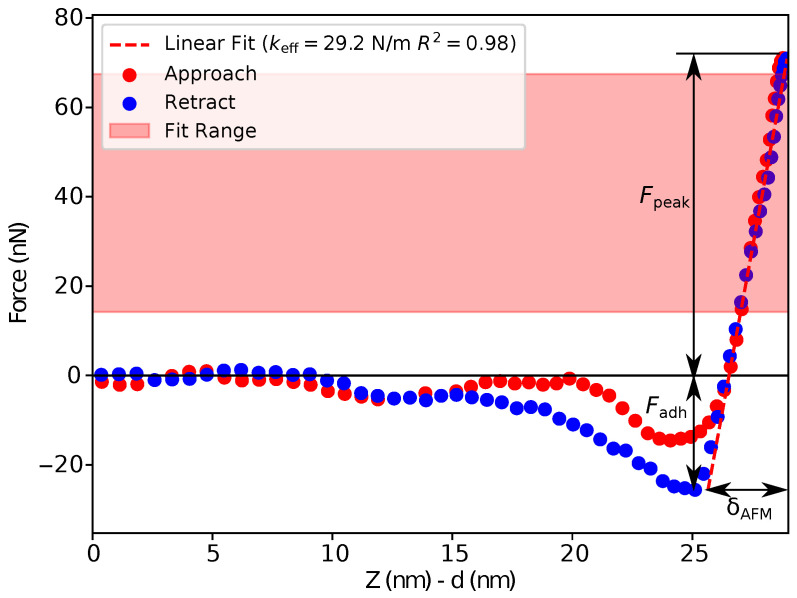
An example of force–distance curve processing with the Reissner model. The pink rectangle shows the user-defined fitting region of the force–distance curve. $F_{\mathrm{peak}}$ is the maximum applied force to the silica hollow particle, $F_{\mathrm{adh}}$ is the adhesion force between the probe and the particle, $\delta_{\mathrm{AFM}}$ is the total deformation of the shell by the probe and rigid substrate. The effective (i.e., the deformation of the hollow particle by the rigid substrate is neglected) stiffness of the shell ($k_{\mathrm{eff}}$ in the legend) was calculated as a slope of the force–distance curve in the fitting region (the red dashed line represents the result of the linear fit), $R^{2}$ in the legend is the coefficient of determination of the linear regression.

**Figure 3:**
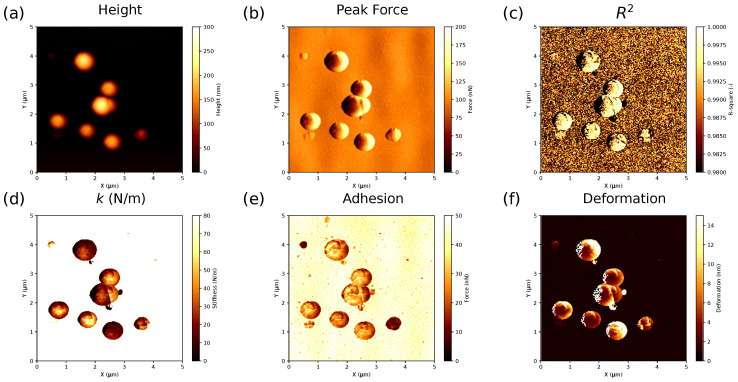
(**a**) AFM height, (**b**) peak force $F_{\mathrm{peak}}$, (**c**) coefficient of determination $R^{2}$, (**d**) effective stiffness *k*, (**e**) adhesion $F_{\mathrm{adh}}$ and (**f**) total deformation $\delta_{\mathrm{AFM}}$ maps. Scan size is $5\times 5\;\mu m^{2}$ for all the images.

The general processing workflow consists of several subsequent steps:

1. The extraction of the raw data and division of the data array to 65,536 pairs (approach and retract) of the force–displacement curves for the AFM image with a resolution of 256×256 pixels using the Sync. Distance value evaluated at calibration.
2. The recalculation of the force–displacement curves as the AFM cantilever deflection (*d* in nm instead of in V in the raw data) vs. the Z-piezo position (*Z* in nm instead of time in 2μs time steps) using the calibration results.
3. The baseline correction. Force–displacement curve measurements may be affected by optical interference, thermal drift, electrostatic long-range forces, hydrodynamic force due to viscous friction of the cantilever, etc. As a consequence, a non-zero cantilever deflection is measured in the pre-contact region between the probe and the sample surface. Thus, a proper baseline correction should be performed before the data analysis. In our program, an option allows the user to select a number of points defining the baseline. Afterwards, a linear baseline was calculated using linear regression in the user-selected non-contact range for each force–displacement curve and subtracted from the initial data.
4. Correction for the cantilever bending to convert the Z-piezo position (*Z* in nm) to distance (Z−d in nm) by subtraction of the cantilever deflection *d*.
5. Fitting the curves using a selected contact mechanics model (e.g., the Hertz, DMT, JKR, Reissner, Berry model) in the user-defined fitting region of the force–distance curve (Figure 2) and visualization of the resolved spatial map of the Young’s modulus, local stiffness, indentation, adhesion force, etc. depending on the model used (Figure 3). The coefficient of determination of the linear regression $R^{2}$ for the approximation of the experimental data by the model used was also calculated for each force–distance curve to estimate the quality of the fit.

### 2.5. Contact Mechanics Models for Small Elastic Deformations of Hollow Spheres

The Hertz and Reissner theories are generally used for the evaluation of Young’s modulus from force–deformation AFM curves for two extreme limits of the spherical hollow particles, i.e., homogeneous solid particles (the shell thickness $h_{s}$ is equal to the particle radius *R*) and thin-shelled hollow particles ($h_{s}\ll R$), respectively.

In the Hertz theory, small (compared to the radii of the interacting spheres) elastic deformations at the contact of two homogeneous isotropic solid spheres with radius $R_{s}$ and $R_{p}$, with a Young’s modulus of $E_{s}$ and $E_{p}$, and the Poisson’s ratio of $\nu_{s}$ and $\nu_{p}$ are analysed. The force *F* acting between the spheres is related to their total indentation δ by:

(1) $$F=\frac{4}{3}E^{*}{\bar{R}}^{1/2}\delta^{3/2},$$

where the reduced Young’s modulus $E^{*}$ is

(2) $$\frac{1}{E^{*}}=\frac{1-\nu_{s}^{2}}{E_{s}}+\frac{1-\nu_{p}^{2}}{E_{p}},$$

and the effective radius $\bar{R}$ is

(3) $$\frac{1}{\bar{R}}=\frac{1}{R_{s}}+\frac{1}{R_{p}}.$$

If the Young’s modulus of the AFM probe $E_{p}$ is much greater than that of the particle $E_{s}$ then:

(4) $$F=\frac{4E_{s}{\bar{R}}^{1/2}}{3(1-\nu_{s}^{2})}\delta^{3/2},$$

otherwise the reduced Young’s modulus (2) must be used for the analysis.

In AFM experiments, the particles under study are usually immobilized on a rigid substrate. Thus, the compression of the particles leads to indentation at both the top of the particle by the AFM probe ($\delta_{\mathrm{eff}}$) and at the bottom by the substrate ($\delta_{\mathrm{subs}}$). Correction factors that account for the substrate indentation of the particle in the Hertz model have been derived analytically by Glaubitz et al. [76]:

(5) $$C=\frac{\delta_{\mathrm{eff}}}{\delta_{\mathrm{afm}}}=\frac{{\left[\left(R_{p}/R\right)+1\right]}^{1/3}}{{\left[\left(R_{p}/R\right)+1\right]}^{1/3}+{\left(R_{p}/R\right)}^{1/3}},$$

where $\delta_{\mathrm{afm}}=\delta_{\mathrm{eff}}+\delta_{\mathrm{subs}}$ is the total indentation measured in the AFM experiments.

The Hertz theory does not account for the adhesion force between the AFM probe and the particle, strongly affecting the results of the measurements in AFM because of the small contact radius. To incorporate the effect of adhesion into the Hertzian contact geometry, various contact mechanics models (e.g., the Derjaguin–Muller–Toporov (DMT), Johnson–Kendall–Roberts (JKR), and Maugis–Dugdale models) have been developed. For example, in the DMT model, which is used in the PeakForce QNM for real-time analysis of the force–distance curves, the contact profile is assumed to be the same as in the Hertz theory but with an additional adhesion force $F_{\mathrm{adh}}$ independent of the contact area:

(6) $$F=\frac{4E_{s}{\bar{R}}^{1/2}}{3(1-\nu_{s}^{2})}\delta^{3/2}+F_{\mathrm{adh}}.$$

The assumption of homogeneity of the interacting spheres in the Hertz model is obviously not valid for hollow particles. For analysis of small elastic indentations in thin-shelled hollow particles, the Reissner model is generally used. In this model, the force *F* is related to the indentation δ as:

(7) $$F=\frac{4E_{s}h_{s}^{2}}{R\sqrt{3(1-\nu_{s}^{2})}}\delta ,$$

where *R* is the particle radius, $h_{s}$ is the shell thickness, $E_{s}$ and $\nu_{s}$ are the Young’s modulus and Poisson’s ratio of the shell material, respectively. The application of (7) is appropriate for hollow particles with a shell thickness to particle radius ratio $h_{s}/R<1/10$ and indentations δ less than the shell thickness $h_{s}$.

The Hertz (4) and Reissner (7) theories are analytical solutions for small deformations at two extreme limits of the shell thickness to capsule radius ratio $h_{s}/R$, i.e., $h_{s}/R\ll 1$ for the Reissner model and $h_{s}/R=1$ for the Hertz model. For hollow spheres with arbitrary $h_{s}/R$ ratio immobilized on a rigid substrate and deformed with a probe of $R_{p}$ radius, Berry et al. reported the results of numerical simulations and suggest the following generalized equation [77]:

(8) $$\frac{F\sqrt{3(1-\nu_{s}^{2})}}{{\left(h_{s}/R\right)}^{3}4R^{2}}=E_{s}\beta {\left(\frac{C\delta_{\mathrm{afm}}}{h_{s}}\right)}^{\alpha },$$

where the correction factor $C=\delta_{\mathrm{eff}}/\delta_{\mathrm{afm}}$, accounting for the effect of a rigid substrate on particle indentation, coefficient β and scaling exponent α are free model parameters depending on the shell thickness to particle radius ratio $h_{s}/R$, the AFM tip radius to particle radius ratio $R_{p}/R$, and to a lesser extent the Poisson’s ratio of the shell material $\nu_{s}$. The Reissner solution ($h_{s}/R\ll 1$) gives α=1 and β=1, the Hertz solution ($h_{s}/R=1$) yields α=3/2 and $\beta ={\left(\bar{R}/R\right)}^{1/2}/\sqrt{3(1-\nu_{s}^{2})}$. The values of the parameters α, β and *C* were evaluated by Berry et al. using numerical simulations and are collected in the supplementary information (Table S1) in their article [77]. Thus, Equation (8) represents a family of contact mechanic models with various power-dependence of force *F* on the total indentation measured by AFM $\delta_{\mathrm{afm}}$, i.e., $F∼\delta_{\mathrm{afm}}^{\alpha }$, where the scaling exponent α varies in the range from 1 (the Reissner solution for thin shells) to 1.5 (the Hertz solution for uniform particles).

To use the results of the numerical simulations for evaluation of the Young’s modulus one needs to measure the values of the particle (*R*) and AFM tip ($R_{p}$) radii, and estimate the shell thickness $h_{s}$ and Poisson’s ratio $\nu_{s}$ of the shell material. Following Zhang et al. [45,46], the Poisson’s ratio was assumed to be $\nu_{s}=0.17$, i.e., that of fused silica. The shell thickness $h_{s}$ was evaluated by TEM. The value of the AFM tip radius was determined according to the procedure described in Section 2.4.2. The radius of each individual hollow particle (*R*) was measured by AFM (as a half of the particle height) using the lowest scanning peak force to minimize its deformation. Then, using the values of $\nu_{s}$, $h_{s}/R$ and $R_{p}/R$, the coefficients α, β and *C* of the Berry et al. model (8) were evaluated. After this, the approach part of the force–distance curves was linearized in the coordinates ${\left(F-F_{\mathrm{adh}}\right)}^{-\alpha }$ vs. $\delta_{\mathrm{afm}}$, and the slope of the curve *k* and the coefficient of determination $R^{2}$ of the fit were evaluated by linear regression for each force–distance curve in the AFM image.

## 3. Results and Discussion

**Figure 4:**
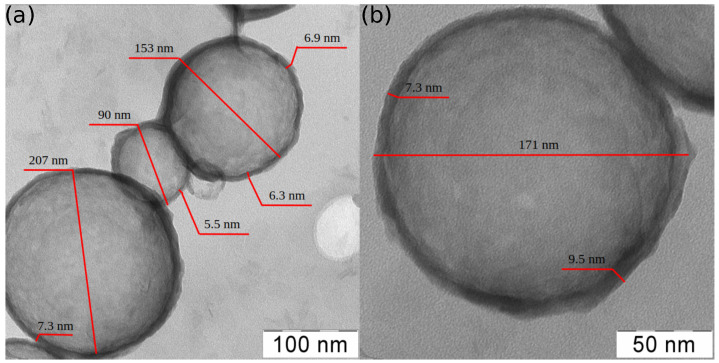
(**a**) TEM image of the silica hollow particles of different diameter and shell thickness. (**b**) TEM image of an individual silica particle showing variation in the shell thickness.

Figure 4 shows representative TEM images of the silica hollow particle under study. As one can see from the TEM images, the particles have spherical shape with notable size polydispersity. They appear translucent, and the shell thickness can be estimated from the width of the dark rims around each particle. The mean shell thickness was $h_{s}=8\pm 2$ nm without notable dependence on the particle size (the mean shell thickness and its standard deviation were evaluated using TEM images of 35 silica particles). However, one can observe some variations in the shell thickness of different particles as well as non-ideal uniformity of the shell thickness.

**Figure 5:**
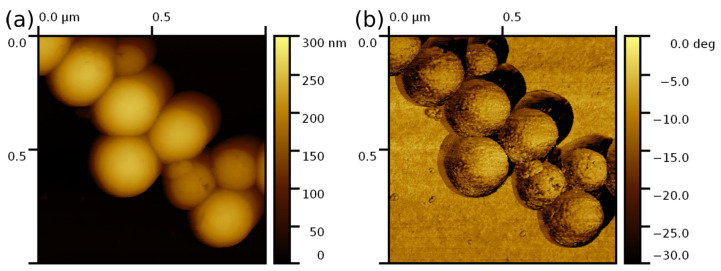
AFM (**a**) height and (**b**) phase images of the silica hollow particles scanned in the Tapping mode with a sharp commercial RTESP-300A probe (Bruker, San Jose, CA, USA). Scan size is $1\times 1\;\mu m^{2}$. The RMS roughness of the shell surface estimated on an area of about $100\times 100\;{\mathrm{nm}}^{2}$ is $R_{q}=1.0\pm 0.3$ nm.

To estimate surface roughness of the shell, we investigated the surface topography by AFM in the Tapping mode using sharp commercial probes RTESP-300A (Bruker, San Jose, CA, USA). The shell surface has granular morphology and appears to be composed of small silica particles chemically bounded together (Figure 5b). The root-mean-square roughness of the shell surface was estimated as $R_{q}=1.0\pm 0.3$ nm using AFM images of 15 particles, similar to that of the as-synthesized micron-size silica hollow spheres prepared by the Stöber method using a polystyrene bead template (i.e., $R_{q}=1.9\;\mathrm{nm}$ [46]).

**Figure 6:**
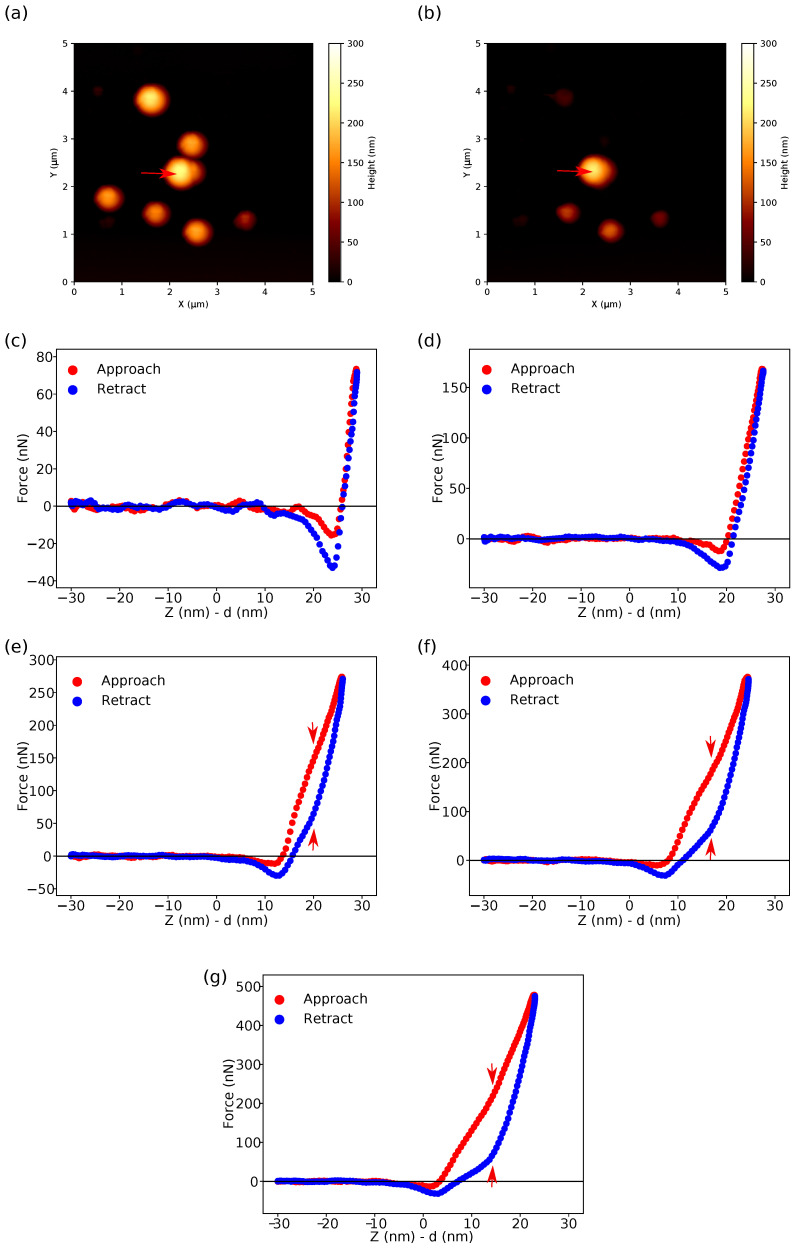
AFM height images scanned in the PeakForce QNM mode with a “dull” tip at the peak force of $F_{\mathrm{peak}}$ of (**a**) 100 and (**b**) 500nN. The scan size is $5\times 5\;\mu m^{2}$. The force–distance curves recorded at the “north” pole of the hollow silica particle are marked with a red arrow in the height images at $F_{\mathrm{peak}}$ of (**c**) 100, (**d**) 200, (**e**) 300, (**f**) 400, and (**g**) 500 nN. The red arrows in the subfigures (**e**–**g**) indicate the inflection points on the force–distance curves.

Figure 6 shows AFM height images of the silica particles immobilized on an APTES-modified silicon wafer (Figure 6a,b) and typical force–distance curves recorded in the PeakForce QNM mode with a “dull” tip on the top (i.e., at the “north” pole) of the particle marked with a red arrow in the height images with progressively increasing peak force $F_{\mathrm{peak}}$ from 100 to 500 nN (Figure 6c–g). It should be noted that some of the hollow particles were ruptured or detached from the substrate by scanning at high applied forces (cf. Figure 6a,b). As one can see in Figure 6c,d, the loading and unloading force–distance curves coincide at low applied forces (i.e., $F_{\mathrm{peak}}<200$ nN) without notable hysteresis between the curves. The force is linearly dependent on the deformation in accordance with the Reissner model for elastic deformations of thin spherical shells. However, at higher forces (cf. Figure 6e–g), the loading force–distance curves start with a linear section followed by a non-linear region with progressively decreasing slope which develops into an “inflection point” (marked with a red arrow on the plots) and finally the slope starts to increase again. Along with non-linear behaviour, the force–distance curves measured at high applied forces reveal pronounced hysteresis between the loading and unloading curves increasing with the applied force (cf. Figure 6e–g). It should be noted that the change in the slope on the unloading curves and the “inflection point” on the loading curves can be observed at about the same values of deformation. Thus, one can observe the elastic response of the shells in only a rather limited range of the applied forces.

The observed behaviour is consistent with other studies on spherical shell systems [20,21,45,54], showing that for deformations of the order of or slightly higher than the shell thickness the linear elastic dependency was lost and further deformation led to buckling instability, i.e., the formation of a region of reversed curvature (a “dimple”) which grows with increasing deformation. This type of deformation is energetically favoured, since in this way thin shells confine the energetically “high-cost” shell stretching to the fold region, i.e., the rim of the “dimple”, while the “low-cost” shell bending dominates elsewhere. Under the assumption that the deformation energy is localized on the rim of the “dimple”, Pogorelov derived the following result for the deformation δ of thin spherical shells under the point load *F* [88]:

(9) $$\delta =\frac{{\left(1-\nu_{s}^{2}\right)}^{2}}{3.56}\frac{F^{2}R^{2}}{E^{2}h_{s}^{5}},$$

where *E* and $\nu_{s}$ are the Young’s modulus and Poisson’s ratio of the shell material, respectively, and *R* and $h_{s}$ are the particle radius and shell thickness, respectively. In contrast to the Reissner model (7) for small deformations of the shell, after buckling and formation of a dimple, the deformation is no longer linear with the force, but quadratic (9), explaining the progressively decreasing slope of the force–distance curves right after the linear region (cf. Figure 6e–g). The Pogorelov regime of shell deformation is limited by the elastic response of the shell material [88]. When the dimple grows with increasing deformation, the mechanical stress in the shell localized on the rim of the dimple also increases [88]. After the stress reaches the yield stress of the shell material, further growth of the dimple leads to very large energy deformation, fixing the dimple geometry and resulting in a sharp increase in the slope of force–distance curves (cf. Figure 6e–g). The hysteresis between the loading and unloading force–distance curves increasing with the applied force (cf. Figure 6e–g) also indicates the existence of irreversible processes such as plasticity above a certain deformation level [54]. A similar force response of elastic–plastic microcapsules under compression, including successive softening and stiffening of the force response at large deformations beyond the elastic regime, was predicted using finite element modelling and confirmed with micromanipulation compression experiments [89,90].

**Figure 7:**
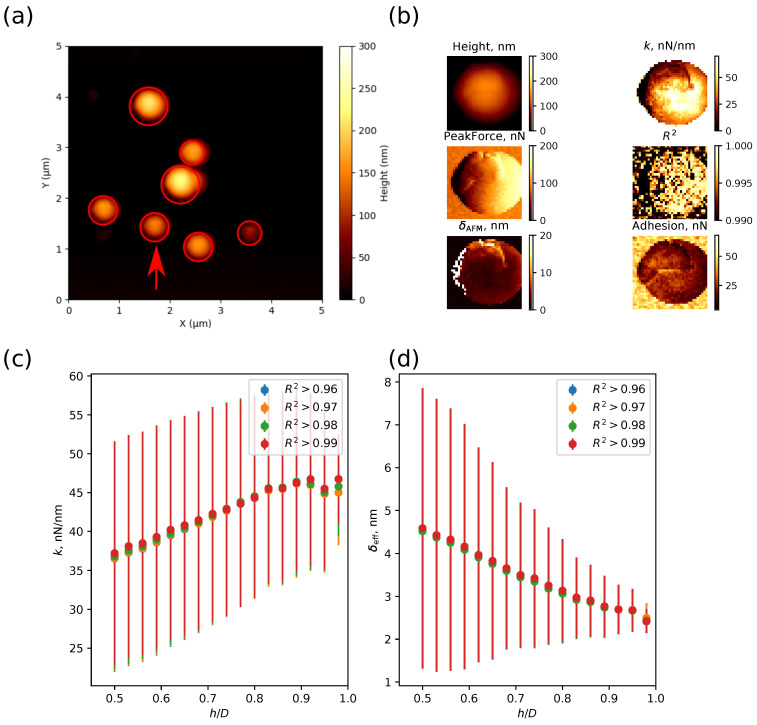
(**a**) AFM height image of the hollow silica particles scanned at the peak force of $F_{\mathrm{peak}}=100$ nN. (**b**) The height, peak force, total deformation $\delta_{\mathrm{AFM}}$, effective stiffness *k*, coefficient of determination of the fit $R^{2}$ and adhesion maps for the particle are marked with a red arrow in the height image. The size of the images is $600\times 600\;{\mathrm{nm}}^{2}$. (**c**) Average effective stiffness *k* and (**d**) effective deformation of the particle by the AFM probe $\delta_{\mathrm{eff}}$ calculated for the pixels of the AFM image where h/D ratio, i.e., the ratio of the height at the pixel to the particle diameter, is higher than the threshold value in the range from 0.5 to 1 and the linear regression coefficient of determination $R^{2}$ is greater than 0.96, 0.97, 0.98, and 0.99, respectively.

Figure 7 illustrates the effect of a local surface slope on the accuracy of the mechanical measurements by AFM. As one can see in Figure 7b, the larger the distance between the point on the particle surface and its “north pole”, the greater the estimated value of the deformation (cf. the $\sigma_{\mathrm{AFM}}$-map where the particle centre appears darker than the rim), and the lower the value of the effective stiffness (cf. the *k*-map where the particle centre appears brighter than the rim). This effect can be explained by the existence of lateral forces acting on the AFM tip at non-vertical contact between the tip and the sample, resulting in additional contributions to the normal deflection of the cantilever and incorrect evaluation of the deformation. The lateral forces are dependent on the AFM probe geometry (the cantilever length and tip height), the local slope of the sample and the type of tip–sample contact [67]. Inaccurate evaluation of the deformation leads to an error in estimating the normal contact stiffness and elastic properties of the sample. This effect is demonstrated in Figure 7c,d, where the local surface slope is evaluated using the h/D parameter, i.e., the ratio of the height *h* in a point on the particle surface relative to the substrate to particle diameter *D* (h/D=1 for the north pole). As one can see, the average value of the effective deformation $\delta_{\mathrm{eff}}$ and its standard deviation decreases with an increase in h/D, reaching a plateau value at h/D>0.95 (Figure 7d). It should be noted that variation in the $R^{2}$ threshold, reflecting the quality of the force–distance curve fit with a chosen contact model, ranging from 0.96 to 0.99 has little effect on the value of the effective deformation. A similar effect of h/D and $R^{2}$ thresholds on the effective stiffness of the shell *k* is shown in Figure 7c. To minimize the effect of local surface slope, only the points on the particle surface with h/D>0.95 and $R^{2}>0.96$ were used for further analysis.

**Figure 8:**
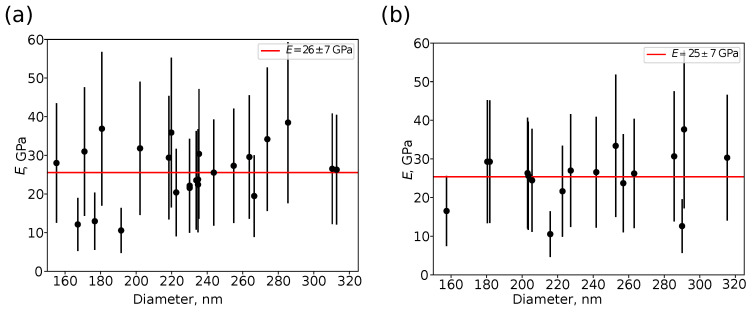
The Young’s modulus of the shell *E* as a function of particle diameter measured at the peak force $F_{\mathrm{peak}}$ of (**a**) 100, and (**b**) 200 nN.

Figure 8 shows the dependence of the Young’s modulus of the shell *E* as a function of the particle diameter, evaluated using the Berry et al. model (8), accounting for the effects of finite shell thickness $h_{s}$ and deformation of the particle by a rigid substrate. The shell thickness was taken as $h_{s}=8$ nm based on the TEM data, the radius of an individual particle *R* was evaluated from an AFM height image scanned at the peak force value of $F_{\mathrm{peak}}=100$ nN. The value of the Poisson’s ratio of the shell material was assumed to be $\nu_{s}=0.17$, i.e., the Poisson’s ratio of fused silica [45,46]. The values of α, β and *C* coefficients in (8) were evaluated using the data from the supplementary information in [77]. The Young’s modulus of the shell was found to be independent of the particle diameter (Figure 8). In addition, the mean value of *E* was the same (i.e., 26±7 and 25±7 GPa) in the limit of experimental data scattering for measurements at the peak force $F_{\mathrm{peak}}$ of 100 and 200 nN (the lower number of points in Figure 8b is due to detachment of some hollow silica particles from the substrate). Large error bars in Figure 8 can be explained by two reasons. The first is the variation in local stiffness even for points near the north pole (cf. Figure 7c), related to the inhomogeneity of the shell thickness and variation in the tip–sample contact due to the finite roughness of the shell surface. The second is the variability in the shell thickness of different hollow silica particles, which is impossible to estimate based on the AFM measurements only. The evaluated Young’s modulus of the silica shell E=26±7 GPa is comparable with the modulus of monodisperse hollow silica spheres with a diameter *D* of 790±70, and 1870±60 nm and a shell thickness in the range from 15 to 70 nm reported by Zhang et al. [45], i.e., E=18±6 GPa. That hollow silica spheres were synthesized from tetraethoxysilane (TEOS) by the Stöber method using monodisperse polystyrene particles as a template with its subsequent thermal decomposition. The authors also mentioned that in their study, shells thinner than 15 nm were irreversibly deformed by capillary forces during drying. However, it should be noted that for isotropic compression of ideal hollow spherical particles with a thin uniform shell, the critical pressure resulting in buckling instability $P_{c}$ can be evaluated as [20,21,91]:

(10) $$P_{c}=\frac{2E}{\sqrt{3(1-\nu_{s})}}\frac{h_{s}^{2}}{R^{2}},$$

where $h_{s}$ is the shell thickness, *R* is the particle radius, and *E* and $\nu_{s}$ are the Young’s modulus and Poisson’s ratio of the shell material, respectively. Thus, when particles with a given shell thickness are considered, smaller particles are more robust than larger ones under isotropic compression, e.g., during drying. The hollow silica particles studied in our work have a notably thinner shell ($h_{s}\approx 8$ nm) but also smaller size (D≈200–300 nm). A small diameter results in an increase in the systematic error in the evaluation of the mechanical properties of the shell for non-axisymmetric contact between the AFM tip and sample due to the local surface slope effect. On the other hand, a decrease in the particle size at fixed shell thickness leads to enhanced critical pressure preventing their buckling and rupturing induced by capillary forces while drying. Similar values of the Young’s modulus were reported by Peukert et al. [48,49], who studied the mechanical properties of individual spherical colloidal solid silica particles synthesized by the Stöber–Fink–Bohn method, i.e., E=32.4±6.1 GPa. It is noteworthy that the Young’s modulus of the as-synthesized hollow silica particles and solid silica particles is significantly lower than that of fused silica, i.e., E=76 GPa. However, annealing of the hollow [46] and solid [48,50] silica particles at temperatures above $1000{\;}^{\circ }C$ caused a strong increase in their Young’s modulus to the values typical for bulk-fused silica. The difference in the mechanical properties between the as-synthesized and annealed silica particles was attributed to their internal structure, i.e., lower density and overall reduced silica network connectivity of the as-synthesized particles. This assumption was supported by the solid-state NMR measurements [46,48,50]. Zhang et al. reported that the hollow silica particles synthesized using hyperbranched PEG-PEOS precursor consist of a mesoporous shell with a pore diameter of 2–4 nm [3,52]. Thus, one can suggest that porosity of the shell could explain the relatively low Young’s modulus compared to that of fused silica. However, this value is comparable with that of the as-synthesized hollow and solid silica particles reported in the literature [45,46,48,49,50].

**Figure 9:**
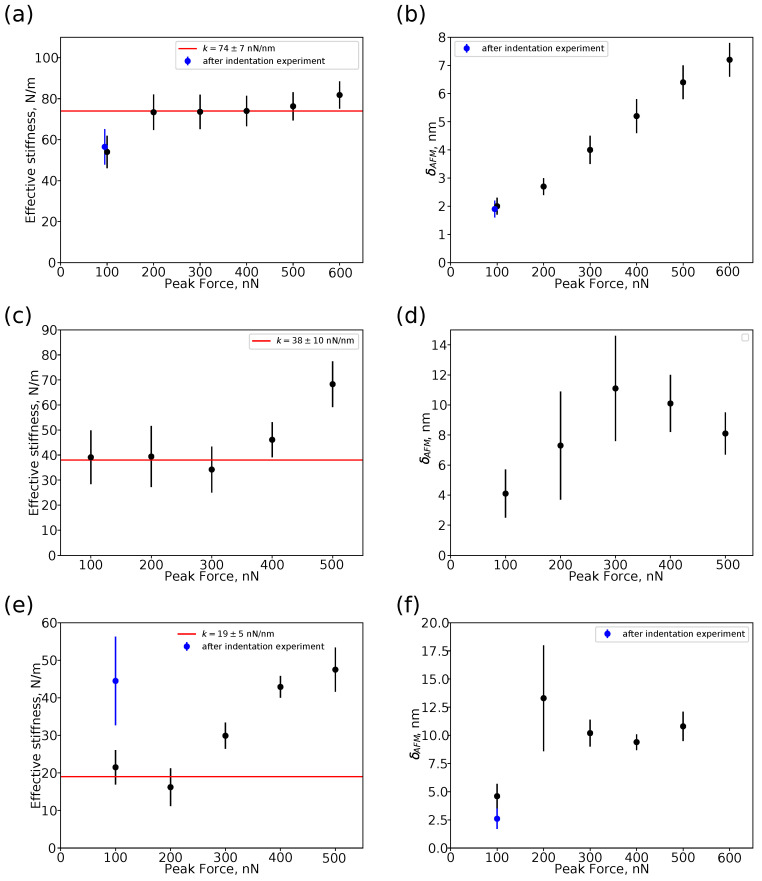
Effective stiffness $k_{\mathrm{eff}}$ (**a**,**c**,**e**) and total deformation $\delta_{\mathrm{AFM}}$ (**b**,**d**,**f**) of the shell as a function of the applied peak force for the particles with effective stiffness of 74±7 N/m (**a**,**b**), 38±10 N/m, (**c**,**d**), and 19±5 N/m (**e**,**f**).

As mentioned above, some silica particles were detached from the substrate during the scanning even at relatively low applied forces (e.g., 200 nN). To investigate the mechanical behaviour of hollow silica particles under high applied loads beyond the elastic deformation limits, the particles were partially embedded in a thin polystyrene film obtained by spin coating on a silicon wafer. It was found that at low applied forces, the mechanical response of the particles was independent of the immobilization method, in line with the previous observations of Zhang et al. [46]. Variation in shell thickness between different particles results in significant variation in their stiffness (cf. Figure 8), and as a consequence their different behaviour under high applied forces. Figure 9 shows the plots of effective stiffness $k_{\mathrm{eff}}$ (Figure 9a,c,e) and total deformation $\delta_{\mathrm{AFM}}$ (Figure 9b,d,f) as a function of applied peak force $F_{\mathrm{peak}}$ for the hollow particles of “high” $k_{\mathrm{eff}}=74\pm 7$ N/m (Figure 9a,b), “medium” $k_{\mathrm{eff}}=38\pm 10$ N/m (Figure 9c,d), and “low” $k_{\mathrm{eff}}=19\pm 5$ N/m (Figure 9e,f) stiffness. The peak force $F_{\mathrm{peak}}$ was progressively increased from 100 to 500 nN, afterwards the samples were scanned at $F_{\mathrm{peak}}=100$ nN again to check reversibility of the particle geometry and its mechanical response (blue points in Figure 9).

**Figure 10:**
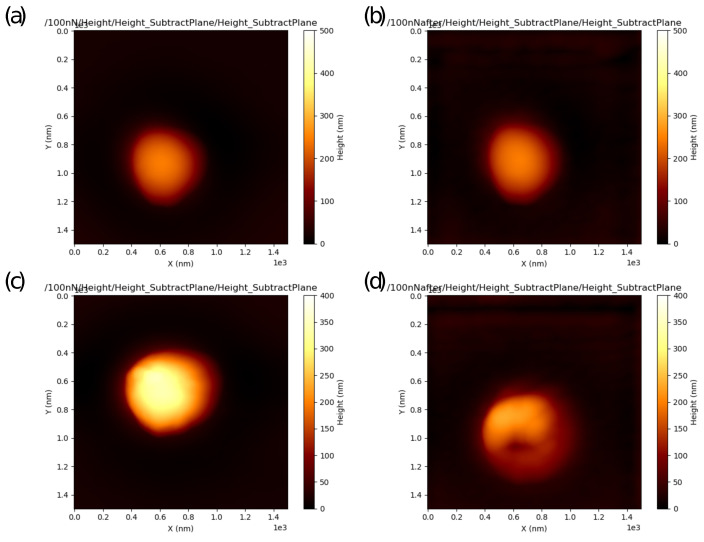
AFM height images scanned at $F_{\mathrm{peak}}=100$ nN recorded before (**a**,**c**) and after (**b**,**d**) scanning in the PeakForce QNM mode with progressively increasing $F_{\mathrm{peak}}$ in the range from 100 to 500 nN. (**a**,**b**) The “stiff” hollow particle (k=74±7 N/m) with elastic response in the whole range of $F_{\mathrm{peak}}$, (**c**,**d**) the “soft” hollow particle (k=19±5 N/m) with irreversible deformation of the shell.

For the particle of “high” stiffness (Figure 9a,b), the effective stiffness $k_{\mathrm{eff}}$ is independent of $F_{\mathrm{peak}}$ in the range from 200 to 500 nN. Lower values of $k_{\mathrm{eff}}$ at $F_{\mathrm{peak}}=100$ nN can be explained by low shell deformation by the AFM probe ($\delta_{\mathrm{eff}}$ is about 1 nm, comparable with the shell surface roughness). A similar so-called “skin effect” was reported earlier for very small (<2 nm) indentations of soft polymeric materials [60] and rigid inorganic materials (e.g., fused silica, graphite, silicone, gold) [63]. Possible explanations of this effect include non-linearity of the stress–strain relation and asperities on the tip (or sample) apexes resulting in non-correct evaluation of the tip–sample contact area and, as a consequence, an error in determination of the Young’s modulus value. The total deformation of the particle by the AFM probe and rigid substrate $\delta_{\mathrm{AFM}}$ increases nearly linearly with the peak force $F_{\mathrm{peak}}$ in accordance with the Reissner model for elastic deformations of thin-shelled hollow particles. The final scanning at $F_{\mathrm{peak}}=100$ nN demonstrates that the particle retains its spherical shape (cf. Figure 10a,b) and mechanical properties (cf. blue symbols in Figure 9a,b), i.e., the shell deformations were reversible.

For the particle of “medium” stiffness, the effective stiffness $k_{\mathrm{eff}}$ is about the same at $F_{\mathrm{peak}}=100$ and 200 nN, after that it slightly decreases at $F_{\mathrm{peak}}=300$ nN, and then sharply increases at $F_{\mathrm{peak}}=400$ and 500 nN (Figure 9c). Such behaviour indicates the transition from reversible elastic deformations of the shell at low applied forces to buckling instability and irreversible deformations, where a slight decrease of $k_{\mathrm{eff}}$ is related to the decreasing slope of the force–distance curves right after the elastic linear region (i.e., the Porgorelov regime of the shell deformation) followed by a sharp increase in this slope due to the plasticity effect (Figure 6e–g). The total deformation $\delta_{\mathrm{AFM}}$ increases with applied peak force in the range from 100 to 300 nN, and then slightly decreases (Figure 9d). This decrease is associated with residual deformation of the silica shell.

For the “soft” particle, a slight decrease in the effective stiffness of the shell $k_{\mathrm{eff}}$ can be already observed at $F_{\mathrm{peak}}=200$ nN, after this the evaluated effective stiffness increases with the peak force $F_{\mathrm{peak}}$ (Figure 9e). As for the total deformation $\delta_{\mathrm{AFM}}$, its value increases in the peak force range from 100 to 200 nN, then slightly drops at $F_{\mathrm{peak}}=300$ nN, and remains nearly constant at higher forces (Figure 9f). The final scanning at $F_{\mathrm{peak}}=100$ nN indicates an irreversible change in the mechanical response, the effective stiffness stays at about constant at $F_{\mathrm{peak}}=500$ nN, whereas the total deformation is about two times lower compared to the initial deformation at the first scanning at $F_{\mathrm{peak}}=100$ nN (cf. the blue symbols in Figure 9e,f). An irreversible deformation of the particle shell can also be observed in the AFM height images (Figure 10c,d). The hollow silica particle was spherical before the indentation experiments (Figure 10c), but one can see a dimple on the top of the particle at the final scanning (Figure 10d). Thus, scanning in a wide range of the peak force not only enables evaluation of the Young’s modulus of the shell in the elastic regime of deformations but also estimation of the applied force level and corresponding shell deformation resulting in buckling instability.

**Figure 11:**
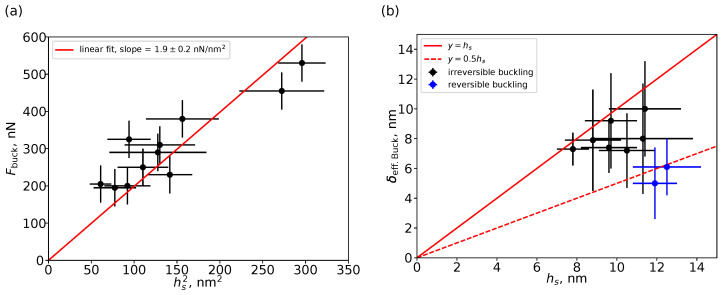
(**a**) The buckling force $F_{\mathrm{buck}}$ as a function of the square of the estimated shell thickness $h_{s}^{2}$. (**b**) The effective shell deformation by the AFM probe resulting in buckling instability $\delta_{\mathrm{eff}}.\mathrm{Buck}.$ as a function of the estimated shell thickness $h_{s}$.

As a criterion of buckling instability, a small decrease in the effective stiffness before its following sharp increase was used (cf. Figure 9c,e). The effective shell deformation in this point reaches its local maximum (cf. Figure 9d,f). The shell thickness value $h_{s}$ was estimated from the effective stiffness of the particle measured in the elastic range of deformations (i.e., at low peak forces), using (8) with α=β=1, i.e., in the Reissner model but accounting for the effect of particle deformation by a rigid substrate. The Young’s modulus of the shell E=26±7 GPa was assumed to be independent of the shell thickness and particle size, and the Poisson’s ratio was taken as $\nu_{s}=0.17$. The particle radius was evaluated from its height in the AFM images scanned at $F_{\mathrm{peak}}=100$ nN. Zhang et al. reported that the buckling force increases as the square of the hollow silica particle shell thickness $h_{s}$ in the range from 15 to 70 nm, but they did not observe the effect of the particle diameter on the value of the buckling force in the studied size range (D=800 and 1900 nm) [45]. We also did not observe the effect of the particle diameter on the buckling force in our study. However, the buckling force notably increased with the estimated shell thickness $h_{s}$. Figure 11a shows plots of the buckling force $F_{\mathrm{buck}}$ as a function of $h_{s}^{2}$. The slope of the $F_{\mathrm{buck}}$ vs. $h_{s}^{2}$ dependence is $1.9\pm 0.2\;\mathrm{nN}\;{\mathrm{nm}}^{-2}$, close to the value evaluated from the data published by Zhang et al., i.e., about $1.5\;\mathrm{nN}\;{\mathrm{nm}}^{-2}$ [45]. Thus, one can suggest that control of the shell thickness in a wide range enables effective adjustment of the buckling force of hollow silica particles.

One can see notable experimental scattering in Figure 11a. A probable reason of this uncertainty is that the buckling transition, i.e., a deviation from the linear regime in the force–distance curves (cf. Figure 6e–g), is not sharply defined. Another reason could be related to the effect of shell imperfections (e.g., non-uniformity in thickness) on its buckling behaviour [91,92,93]. Figure 11b shows the plot of the effective deformation of the shell by the AFM probe resulting in its buckling instability $\delta_{\mathrm{eff}}.\mathrm{Buck}.$ as a function of the estimated shell thickness $h_{s}$. It should be noted that for an ideal spherical shell of constant thickness, the buckling point is encountered at the deformations of the shell thickness [91,94]. However, as one can see in Figure 11b, the buckling instability appears at deformations lower than the estimated shell thickness. The ratio of $\delta_{\mathrm{eff}.\mathrm{Buck}.}$ to $h_{s}$ was varied in the wide range from 0.4 to 1. For most of the hollow silica particles, their geometry and mechanical properties were changed irreversibly after the shell buckling, except for two of the most stiff particles (cf. blue symbols in Figure 11b). Thus, the lower values of the buckling deformations can be explained by imperfection in the shell structure, e.g., non-uniformity in the shell thickness revealed by TEM (Figure 4) and the shell surface roughness revealed by AFM (Figure 5).

## 4. Conclusions

The mechanical properties of hollow silica particles with a diameter of 100–300 nm and a shell thickness of 8±2 nm synthesized using a self-templating amphiphilic PEG-PEOS polymeric precursor were studied using a high-frequency AFM indentation method based on the PeakForce QNM mode. This technique enables simultaneous visualization of the surface morphology and high-resolution mapping of the mechanical properties with a load force resolution up to a few tens of pN and indentation depths as small as 1 nm. These advantages are crucial for measurements of submicron-sized capsule systems because high spatial resolution is needed for precise positioning of the AFM probe relative to the capsule, whereas elastic deformations of the thin-shelled hollow particles are generally limited by their shell thickness. Moreover, inherent polydispersity in capsule size and shell thickness can result in notable stiffness variations and consequently the requirement of multiple measurements to obtain statistically significant results. In this context, the high scanning rate in the PeakForce QNM mode comparable with that in the Tapping mode seems to be beneficial. However, despite these advantages, quantitative measurements of capsule elastic proprieties in the PeakForce QNM mode are still challenging.

In this study, we investigated the ability of the PeakForce QNM AFM mode for quantitative measurements of the elastic properties of silica hollow particles. To avoid non-linearity of force–deformation responses and prevent damage to the shell before buckling occurs, relatively dull AFM probes prepared by thermal annealing of commercial silicon probes were used. The factors affecting the accuracy of the mechanical measurements, such as the local slope of the particle surface, deformation of the particles by a rigid substrate, shell thickness variation, and applied force range, were analysed. Within the linear elastic regime, i.e., at small applied forces (<200 nN), the Young’s modulus of the silica shell was evaluated as E=26±7 GPa, independent of the applied force. This value is comparable with the Young’s modulus of micron-sized hollow silica spheres with notably thicker shells (15–70 nm) synthesized by the Stöber method and measured using classical AFM-based force spectroscopy (E=18±6 GPa) [45,46] and that of submicron Stöber particles measured by the nanoindentation technique with a custom-built micromanipulator integrated in an electron microscope (E=32.4±6.1 GPa) [48,49,50]. It should be noted that this value is much lower than the Young’s modulus of fused silica, i.e., 76 GPa, indicating a lower density of the silica shell. Thus, one can conclude that the mechanical properties of the silica hollow particles prepared by this new synthetic approach based on self-assembly of the amphiphilic polymeric PEG-PEOS precursor under mild conditions are rather high and comparable with that of hollow and solid silica particles fabricated by the traditional Stöber method.

We also studied the mechanical behaviour of the hollow silica particles beyond their elastic deformations. At higher applied forces, the buckling instability was observed as a non-linear force–deformation response and non-reversible deformation of the shell. The force required for buckling was found to increase with the evaluated shell thickness in accordance with previously published results for silica hollow spheres [45,47]. Thus, tailoring the shell thickness can be effectively used to control the mechanical stability of the silica capsules. However, it should be noted that uniformity in the shell thickness is also important. It was found that the ratio of the shell deformation at buckling to the evaluated shell thickness was not constant as expected for an ideal shell but varied in the wide range from 0.4 to 1, probably due to non-uniformity of the shell thickness.

## Data Availability

The original data reported in this study are available from the corresponding author on reasonable request.
